# Effect of an mHealth Intervention on Hepatitis C Testing Uptake Among People With Opioid Use Disorder: Randomized Controlled Trial

**DOI:** 10.2196/23080

**Published:** 2021-02-22

**Authors:** Karli R Hochstatter, David H Gustafson Sr, Gina Landucci, Klaren Pe-Romashko, Olivia Cody, Adam Maus, Dhavan V Shah, Ryan P Westergaard

**Affiliations:** 1 School of Social Work Columbia University New York, NY United States; 2 Department of Medicine School of Medicine and Public Health University of Wisconsin-Madison Madison, WI United States; 3 Department of Population Health Sciences School of Medicine and Public Health University of Wisconsin-Madison Madison, WI United States; 4 Department of Industrial and Systems Engineering Center for Health Enhancement Systems Studies University of Wisconsin-Madison Madison, WI United States; 5 Bureau of Communicable Diseases Wisconsin Department of Health Services Madison, WI United States

**Keywords:** intravenous injections, mHealth, hepatitis C virus, opioid use disorder, mobile phone

## Abstract

**Background:**

The growing epidemic of opioid use disorder (OUD) and associated injection drug use has resulted in a surge of new hepatitis C virus (HCV) infections. Approximately half of the people with HCV infection are unaware of their HCV status. Improving HCV awareness and increasing screening among people with OUD are critical. Addiction-Comprehensive Health Enhancement Support System (A-CHESS) is an evidence-based, smartphone-delivered relapse prevention system that has been implemented among people with OUD who are receiving medications for addiction treatment (MAT) to improve long-term recovery.

**Objective:**

We incorporated HCV-related content and functionality into A-CHESS to characterize the HCV care continuum among people in early remission and receiving MAT for OUD and to determine whether incorporating such content and functionality into A-CHESS increases HCV testing.

**Methods:**

HCV intervention content, including dissemination of educational information, private messages tailored to individuals’ stage of HCV care, and a public discussion forum, was implemented into the A-CHESS platform. Between April 2016 and April 2020, 416 participants with OUD were enrolled in this study. Participants were randomly assigned to receive MAT alone (control arm) or MAT+A-CHESS (experimental arm). Quarterly telephone interviews were conducted from baseline to month 24 to assess risk behaviors and HCV testing history. Cox proportional hazards regression was used to assess whether participants who used A-CHESS were tested for HCV (either antibody [Ab] or RNA testing) at a higher rate than those in the control arm. To assess the effect of A-CHESS on subsets of participants at the highest risk for HCV, additional analyses were performed to examine the effect of the intervention among participants who injected drugs and shared injection equipment.

**Results:**

Overall, 44.2% (184/416) of the study participants were HCV Ab positive, 30.3% (126/416) were HCV Ab negative, and 25.5% (106/416) were considered untested at baseline. At month 24, there was no overall difference in HCV testing uptake between the intervention and control participants. However, among the subset of 109 participants who engaged in injection drug use, there was a slight trend toward increased HCV testing uptake among those who used A-CHESS (89% vs 85%; hazard ratio: 1.34; 95% CI 0.87-2.05; *P*=.18), and a stronger trend was observed when focusing on the subset of 32 participants who reported sharing injection equipment (87% vs 56%; hazard ratio: 2.92; 95% CI 0.959-8.86; *P*=.06).

**Conclusions:**

Incorporating HCV prevention and care information into A-CHESS may increase the uptake of HCV testing while preventing opioid relapse when implemented among populations who engage in high-risk behaviors such as sharing contaminated injection equipment. However, more studies that are powered to detect differences in HCV testing among high-risk groups are needed.

**Trial Registration:**

ClinicalTrials.gov NCT02712034; https://clinicaltrials.gov/ct2/show/NCT02712034

**International Registered Report Identifier (IRRID):**

RR2-10.2196/12620

## Introduction

Prescription and illicit opioid addiction are a growing public health problem in the United States [1]. In 2016, an estimated 2.1 million people had an opioid use disorder (OUD) [2], and more than 47,000 people died from overdosing on opioids [3], killing more Americans each day than motor vehicle car crashes [4]. The growing opioid epidemic and associated injection drug use have also resulted in a surge of new hepatitis C virus (HCV) infections [5-7]. With injection drug use being the primary risk factor for transmission and the leading cause of incidence [8], HCV infection is now the most common chronic blood-borne infection in the United States, surpassing all other nationally notifiable infectious diseases combined [9]. From 2004 to 2014, the incidence rate of acute HCV infection increased by 400%, and admission for opioid injection increased by 622% among young Americans [7].

Effective medications are available for the treatment of both OUD and HCV infection. Medications for Addiction Treatment (MAT) are Food and Drug Administration–approved, evidence-based prescription medications used to assist people in recovery from opioid addiction by relieving withdrawal symptoms and physiological cravings [10]. Despite the availability of these effective treatments for OUD, uptake remains low [11]. Moreover, highly effective, well-tolerated, direct-acting antiviral agents are now available for the treatment of HCV, which provide a sustained virologic response (SVR, ie, a cure) in more than 90% of treated patients in as little as 8 weeks [12-14]. These medications provide hope for eliminating HCV; however, only a small proportion of infected individuals have received treatment [15]. One study that examined HCV treatment uptake among patients who received MAT found that among those aware of their infection, only 14% had received HCV treatment [16]. Exacerbating this problem is the fact that approximately half of the HCV-infected individuals are unaware of their infection [17]. Expanding both addiction and HCV screening, treatment, and prevention services are urgently needed in response to the record number of new HCV infections driven by the opioid crisis.

Addiction treatment regimens vary considerably depending on the substances used, comorbidities, severity of substance use disorder, and individual preferences. However, the most effective treatments for individuals with OUD uniformly include a combination of MAT, counseling, and access to other behavioral health services and social supports [18-20]. The Addiction-Comprehensive Health Enhancement Support System (A-CHESS), developed at the University of Wisconsin, is a smartphone app designed to improve recovery from addiction by offering a bundle of services that address the various challenges often encountered during recovery. A-CHESS provides communication with peers and addiction experts, reminders and alerts to encourage therapeutic goals, educational material, and other support services to patients. In an effort to improve long-term recovery from OUD, our research team conducted a randomized controlled trial (RCT) that paired A-CHESS with MAT [21].

MAT alone is associated with an estimated 50% reduction in the risk of acquiring HCV [22]. Combining MAT with harm reduction information and services may further reduce one’s chances of acquiring HCV. A systematic review that examined the effect of combining MAT with high-coverage needle and syringe programs found an estimated 74% reduction in HCV infection [22]. Our research team previously designed and pilot tested a computerized intervention to reduce risky injection practices and improve screening for HCV among people who inject drugs [23]. The results from this pilot study suggest that the intervention may reduce the harms of sharing of injection equipment and increase HCV screening [24]. We integrated content and functionality from this computerized intervention into A-CHESS to provide people with OUD who are receiving MAT and addiction counseling access to tailored HCV prevention and care resources.

## Methods

### Overall Objectives

The primary goal of the RCT was to assess whether A-CHESS can prevent relapse among people with OUD who are in early remission and receiving MAT. Individuals with diagnosed OUD of at least moderate severity who were receiving addiction treatment at 3 centers in Massachusetts and Wisconsin were randomly assigned in a 1:1 ratio to receive either MAT alone (control arm) or MAT+A-CHESS (experimental arm). A web-based tool was used to generate a sequence of random assignments in blocks of 16 subjects, stratified on recruitment site, gender, and type of MAT. Each assignment was associated with a unique study ID number. The electronic randomization lists were managed by the project director and stored in a secure location in the center’s shared drive. When subjects completed baseline, they received the next available assignment and study ID on the appropriate randomization list, the group assignment was recorded in Research Electronic Data Capture (REDCap) [25], and the site coordinator was informed by phone or email.

We developed and implemented HCV intervention content within the A-CHESS platform to simultaneously evaluate whether A-CHESS improves secondary outcomes related to the HCV care continuum. The objectives of this study were to characterize the HCV care continuum among people in recovery for OUD and to determine whether incorporating HCV-related content and functionality into the A-CHESS system increases HCV testing. Participants were followed for 24 months.

### Overview of the A-CHESS System

A-CHESS contains multiple services designed to address several types of challenges facing people with addiction. Key A-CHESS services include a call for help function, cognitive behavioral therapy boosters, a GPS location tracker for avoiding high-risk locations and finding 12-step meeting locations, games, and audio- and video-based relaxation recording, tailored coping support, and coach-monitored discussion groups. A-CHESS provides a platform for people with OUDs to interact with peers and trained counselors, obtain educational information, and provide participant-level data. These existing features were used to collect data on participants’ HCV risk behaviors and history of HCV care and deliver behavior change interventions tailored to patients’ self-reported stage of HCV care. The study design, recruitment, eligibility, screening process, and addiction-related services incorporated into A-CHESS have been described in the RCT’s published protocol [21].

### HCV Intervention Content and Functionality

The HCV intervention content included dissemination of educational information, private messages tailored to individuals’ stage of HCV care, and a public discussion forum. The educational content is housed within the *Information* tab of A-CHESS and provides answers to HCV-related frequently asked questions and links to factsheets developed by the Centers for Disease Control and Prevention. HCV screening and treatment resources in the individual’s community are provided, along with news articles and videos related to the co-occurring epidemics of opioid injection and infectious diseases.

The private messaging feature of A-CHESS is used by study personnel to send tailored messages related to filling gaps in the HCV care continuum. Private messaging conversations began by delivering participants in each HCV stage of care a standardized message pertaining to their current stage of care. Subsequent messages were then guided by each individual’s unique response but toward the same goal of helping each individual advance appropriately along the HCV care continuum.

A discussion board, named *Staying Healthy*, was also developed, which provides a forum for participants to ask infectious disease–related questions, share HCV treatment experiences, and discuss barriers to testing and treatment. HCV research staff also engage in these conversations to remind A-CHESS users of the importance of being tested for HCV, encourage healthy behaviors, and stimulate discussion related to such topics. A detailed description of the HCV intervention components and functionality has been previously described [26].

### Data Collection

For 2 years, study participants in both the intervention and control arms completed telephone interviews with 1 of 2 study coordinators at baseline and at quarterly intervals (months 4, 8, 12, 16, 20, and 24). At each of these time points, data were collected on illicit opioid use, other nonprescribed substance use, quality of life, retention in treatment, health service use, injection drug use risk behaviors, and HIV and HCV outcomes. Participants were asked whether they had been tested for HCV, the type of test they received (antibody [Ab] test or RNA test), and the date and result of their last test, and if positive, linkage to care and HCV treatment initiation or completion were assessed. A text field, labeled *the comment field*, was available on all surveys for interviewers to enter comments pertaining to the individual’s HCV stage of care if they shared additional details. For example, interviewers use the comment field to record whether the individual was approximating the date of their last HCV test or whether they expressed uncertainty regarding the type of testing they had received. Each phone interview lasted approximately 20 to 30 min. Surveys were identified by a study ID and stored in REDCap [25].

### Characterizing the HCV Care Continuum

To characterize the HCV care continuum for this study population at baseline, participants were assigned 1 of 7 mutually exclusive stages of HCV care using the criteria specified in Table 1 from the baseline survey. These stages take into consideration the type of test received (Ab or RNA test), test result (positive or negative), and how long ago they were tested.

At each quarterly follow-up interview, their stage of HCV care was updated to one of the stages in Table 2 if their survey answers met the specified criteria.

**Table 1:** Criteria for assigning hepatitis C virus stages of care at baseline: Addiction-Comprehensive Health Enhancement Support System Study, 2016-2020.

HCV^a^ stage of care	Criteria for assigning stage of care based on baseline survey answers
HCV untested	If they (1) have never been tested for HCV, (2) do not know whether they have ever been tested, (3) do not know the result of their last HCV test, or (4) it has been over a year since their last HCV RNA negative test^b^
HCV Ab^c^−	If they tested HCV Ab negative in the past year
HCV Ab+ no RNA test	If they ever tested HCV Ab positive and have not received an RNA confirmatory test
HCV Ab+ RNA− (ie, “cleared”)	If they ever tested HCV Ab positive, tested RNA negative in the past year, and have never received HCV antiviral therapy
HCV Ab+ RNA+	If their last RNA test was positive and they have either (1) never received HCV antiviral therapy or (2) received HCV antiviral therapy in the past and either experienced treatment failure or were reinfected
Currently on treatment	If they are currently receiving HCV antiviral therapy
Achieved SVR^d^ (ie, “cured”)	If they received HCV antiviral therapy and have been told by a medical professional that they were successfully cured of HCV

^a^HCV: hepatitis C virus.

^b^Note about criteria (4): if an individual tested RNA negative, spontaneously cleared the virus without treatment, or was cured with treatment over 1 year ago and had not received an RNA negative test in the past year, they were considered HCV untested. This logic was chosen to ensure that the HCV intervention delivered recommendations for retesting to these participants.

^c^Ab: antibody.

^d^SVR: sustained virologic response.

**Table 2:** Criteria for updating hepatitis C virus stages of care at months 4, 8, 12, 16, 20, and 24: Addiction-Comprehensive Health Enhancement Support System Study, 2016-2020.

HCV^a^ stage of care	Criteria for updating HCV stage of care
HCV Ab−	If they were previously considered HCV untested and have tested HCV Ab^b^ negative since the last survey
HCV Ab+ no RNA test	If they tested HCV Ab positive since the last survey and have not received an RNA confirmatory test
HCV Ab+ RNA− (ie, “cleared”)	If they ever tested HCV Ab positive, tested HCV RNA negative since the last survey, and have not received HCV antiviral therapy
HCV Ab+ RNA+	If they tested HCV RNA positive since their last survey and have not received HCV antiviral therapy since the RNA positive test
Currently on treatment	If they are currently receiving HCV antiviral therapy
Achieved SVR^c^ (ie, “cured”)	If they received HCV antiviral therapy and have been told by a medical professional that they were successfully cured of HCV

^a^HCV: hepatitis C virus.

^b^Ab: antibody.

^c^SVR: sustained virologic response.

If none of the conditions in Table 2 were met at the follow-up interview, the individual’s HCV stage remained the same as it was in the previous interview. If interviewers provided any comments in the *comment field* indicating the participant should have been in a stage of HCV care different than this logic assigned, the stage was manually altered accordingly. We have characterized the HCV care continuum for this population by reporting the number of participants in each stage of HCV care at all 7 time points.

### Measures and Statistical Analysis

Baseline descriptive characteristics were assessed to describe the study population in terms of age, age of first opioid use, gender, race or ethnicity, educational attainment, employment status, marital status, HIV comorbidity, mental health conditions, prescribed MAT, and HCV status. Differences in baseline sociodemographic characteristics between the intervention and control groups were assessed using Pearson chi-square or Fisher exact tests for categorical variables and the two-sample unpaired *t* test or Wilcoxon rank-sum test for continuous variables.

The primary outcome of interest was days to any HCV test (Ab or RNA test), which was calculated as the number of days between the date of study enrollment and the first reported Ab or RNA HCV test. Cox proportional hazards regression was used to compare the time to HCV testing uptake between the intervention and control arms. Participants with no reported HCV test who were lost to follow-up were censored at the time of their last quarterly interview, and those who did not receive an HCV test and completed the 24-month interview were administratively censored. As injection drug use is the primary risk behavior driving HCV transmission, 2 additional Cox proportional hazard models were used to assess the effect of A-CHESS on subsets of participants at the highest risk for HCV, including (1) participants who injected drugs and (2) participants who shared injection equipment with another person. Participants were determined to have injected drugs or shared injection equipment if they reported previously (past 4 months) conducting the behavior on the survey, or any survey before, in which they reported HCV testing or were censored. As individuals’ HCV stage of care likely influenced whether they received follow-up testing and how quickly, all analyses adjusted for baseline stage of HCV care, using the stages defined in Table 1. Kaplan-Meier survival curves were created for each risk group to visualize differences in time to HCV testing between intervention and control participants. An intention-to-treat approach was used.

For each time point, we also describe the mean number of days in the previous 4 months that participants in the intervention group used the A-CHESS app. To determine whether engagement with the A-CHESS app was different for higher-risk participants, we conducted negative binomial regression analyses, with cluster robust standard errors to adjust for the nonindependence of observations within persons over time, to compare app use (ie, the number of days they used the app in the 4 months before each survey) between (1) those who injected drugs and those who did not and (2) those who shared injection equipment and those who did not.

All analyses were conducted using Stata version 16 (StataCorp), and statistical significance was determined using α≤.05.

## Results

### Demographic Characteristics

Between April 2016 and April 2018, 416 participants enrolled in the A-CHESS study and completed the baseline survey; 207 were randomly assigned to the control arm and 209 were assigned to receive A-CHESS. Follow-up was completed for the following number of participants: 382.7% (44/416) at month 4, 79.3% (330/416) at month 8, 72.6%, (302/416) at month 12, 70.4% (293/416) at month 16, 61.8% (257/416) at month 20, and 63.9% (266/416) at month 24. There were no significant differences between the initial sample of 416 participants and the final sample of 266 participants (Multimedia Appendix 1).

The baseline descriptive characteristics of the sample are displayed in Table 3. The sample comprised 85.8% (357/416) of non-Hispanic White people and 54.8% (228/416) male participants. The mean age of the participants was 37 (SD 10) years. Of the 416 study participants, 285 (68.5%) had a high school diploma, General Educational Development, or higher degree, and 101 (24.3%) reported being currently employed for wages. Mental health illnesses affected 70.4% (293/416) of the participants, with the most common diagnoses being depression (218/416, 52.4%), anxiety (215/416, 51.7%), posttraumatic stress disorder (121/416, 29.1%), and bipolar or manic depression (108/416, 26.0%). Overall, 0.01% (4/416) of the participants were HIV positive.

The majority of the sample (302/416, 72.6%) was receiving methadone at the time of enrollment, and heroin was the most commonly used opioid. The average age of participants at first opioid use was 21 (SD 7) years, and 39.4% (164/416) of the participants reported that regular opioid use began through a doctor’s prescription. Of the 95 participants who reported using a needle and syringe to inject drugs in the month before study enrollment, 22% (21/95) reported someone else had used their needle after they had used it and 10% (9/95) of the participants reported using a needle after someone else had already used it. There were no significant differences in baseline characteristics between those assigned to the 2 study arms.

**Table 3:** Baseline characteristics, by intervention group (N=416): Addiction-Comprehensive Health Enhancement Support System Study, 2016-2020.

Characteristics		Category	Control (n=207)	Intervention (n=209)	*P* value
**Enrollment site, n (%)**					
	Wisconsin	N/A^a^	2 (1)	0 (0)	—^b^
	Massachusetts Clinic 1	N/A	5 (2.4)	4 (1.9)	—
	Massachusetts Clinic 2	N/A	200 (96.6)	204 (97.6)	—
Age (years), mean (SD)		N/A	37.0 (9.9)	37.4 (10.2)	.73
Age of first opioid use (years), mean (SD)		N/A	21.1 (7.5)	20.5 (6.9)	.45
**Gender, n (%)**					
	Male	N/A	114 (55.1)	114 (54.5)	.91
	Female	N/A	93 (44.9)	95 (45.5)	—
**Race, n (%)**					
	White	N/A	190 (91.8)	200 (95.7)	.10
	Non-White	N/A	17 (8.2)	9 (4.3)	—
**Ethnicity, n (%)**					
	Non-Hispanic or Latino	N/A	190 (91.8)	187 (89.5)	.46
	Hispanic or Latino	N/A	17 (8.2)	21 (10)	—
**Highest education level, n (%)**					
	Less than high school	N/A	63 (30.4)	68 (32.5)	.75
	High school diploma or General Educational Development	N/A	86 (41.5)	76 (36.4)	—
	Some college or 2-year degree	N/A	50 (24.2)	56 (26.8)	—
	4-year college degree or more	N/A	8 (3.9)	9 (4.3)	—
Currently employed, n (%)		Yes	45 (21.7)	56 (26.8)	.23
**Marital status, n (%)**					
	Single	N/A	82 (39.6)	96 (45.9)	.19
	Has a spouse or partner	N/A	125 (60.4)	113 (54.1)	—
Diagnosed with HIV, n (%)		Yes	2 (1)	2 (1)	>.99
Diagnosed with mental health illness other than substance use disorder, n (%)		Yes	142 (68.6)	151 (72.2)	.41
**Current** **medications for addiction treatment, n (%)**					
	Vivitrol	N/A	12 (5.8)	12 (5.7)	>.99
	Suboxone	N/A	44 (21.3)	46 (22)	—
	Methadone	N/A	151 (72.9)	151 (72.2)	—
**HCV^c^ status, n (%)**					
	HCV Untested	N/A	51 (24.6)	58 (27.8)	.56
	HCV Ab^d^ positive	N/A	87 (42)	91 (43.5)	—
	HCV Ab negative	N/A	69 (33.3)	60 (28.7)	—
Injected drugs, n (%)		Yes	45 (21.7)	50 (23.9)	.60
Shared injection equipment, n (%)		Yes	8 (3.9)	16 (7.7)	.10

^a^N/A: not applicable.

^b^*P* value was not calculated when n=0.

^c^HCV: hepatitis C virus.

^d^Ab: antibody.

### A-CHESS Use

The use of A-CHESS decreased over time. Among participants in the intervention arm who completed the 4-month survey, the mean number of days between baseline and month 4 that A-CHESS was used was 30 days. The mean number of days A-CHESS was used between further time points were as follows: 19 days between months 4 and 8, 17 days between months 8 and 12, 15 days between months 12 and 16, 7 days between months 16 and 20, and 3 days between months 20 and 24. There was no significant difference in the mean number of days A-CHESS was used between (1) those who injected drugs and those who did not (odds ratio [OR] 1.06, 95% CI 0.875-1.37; *P*=.43) and (2) those who shared injection equipment and those who did not (OR 1.08, 95% CI 0.767-1.52; *P*=.66).

### Characterizing the HCV Care Continuum

The number of participants in each stage of the HCV care continuum at all time points is presented in Table 4 (Multimedia Appendix 2 visually presents these results for the selected stages). Overall, 25.5% (106/416) of the study population was considered HCV untested, 30.3% (126/416) were HCV Ab negative, and 44.2% (184/416) were HCV Ab positive at baseline. From baseline to month 24, there was a trend toward a more favorable HCV care continuum overall. The proportion of participants untested at each quarterly interview appeared to decrease over time, from 25.5% (106/416) at baseline to 3.4% (9/266) at month 24. Similarly, the proportion of HCV Ab-positive participants who had not received an RNA test appeared to decrease over time, from 5.3% (22/416) at baseline to 2.3% (6/266) at month 24, and the proportion of participants achieving SVR appeared to increase over time, from 7.5% (31/416) to 18.4% (49/266). In addition, as HCV untested participants underwent HCV testing throughout the duration of the study, the proportion of participants considered HCV Ab-negative increased over time, from 30.3% (126/416) at baseline to 48.9% (130/416) at month 24. There were no significant differences in the number of participants in each stage of HCV care when comparing those who used A-CHESS with those in the control group over time (Multimedia Appendix 3).

**Table 4:** Number of participants in each stage of the hepatitis C virus care continuum at baseline and each follow-up: Addiction-Comprehensive Health Enhancement Support System Study, 2016-2020.

HCV^a^ stage of care	Value n (%)						
	Baseline (n=416)	Month 4 (n=344)	Month 8 (n=330)	Month 12 (n=302)	Month 16 (n=293)	Month 20 (n=257)	Month 24 (n=266)
HCV untested	106 (25.5)	72 (20.9)	59 (17.9)	45 (14.9)	31 (10.6)	17 (6.6)	9 (3.4)
HCV Ab^b^−	126 (30.3)	111 (32.3)	118 (35.8)	122 (40.4)	129 (44)	115 (44.7)	130 (48.9)
HCV Ab+ no RNA test	22 (5.3)	10 (2.9)	6 (1.8)	1 (0.3)	0 (0)	3 (1.2)	6 (2.3)
HCV Ab+ RNA− (ie, “cleared”)	26 (6.3)	26 (7.6)	25 (7.6)	22 (7.3)	26 (8.9)	25 (9.7)	27 (10.2)
HCV Ab+ RNA+	103 (24.8)	85 (24.7)	72 (21.8)	64 (21.2)	55 (18.8)	49 (19.1)	43 (16.2)
Currently on treatment	2 (0.5)	10 (2.9)	9 (2.7)	4 (1.3)	5 (1.7)	3 (1.2)	2 (0.8)
Achieved SVR^c^ (ie, “cured”)	31 (7.5)	30 (8.7)	41 (12.4)	44 (14.6)	47 (16)	45 (17.5)	49 (18.4)

^a^HCV: hepatitis C virus.

^b^Ab: antibody.

^c^SVR: sustained virologic response.

### Effect of A-CHESS on HCV Testing

**Figure 1:**
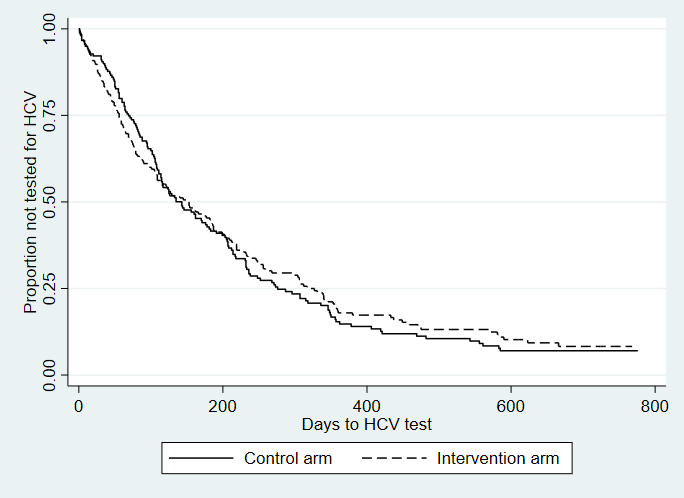
Time to hepatitis C virus test among all study participants, by randomization group: Addiction-Comprehensive Health Enhancement Support System Study, 2016-2020. Hazard ratio: 1.01; 95% CI 0.807-1.26; *P*=.95; n=364. HCV: hepatitis C virus.

Among the 364 participants who completed at least one follow-up interview by month 12, 66.2% (241/364) had received any (Ab or RNA) HCV test. By month 24, 86.3% (314/364) had been tested for HCV. There was no difference in receipt of an HCV test between intervention and control participants overall, where 66.0% (122/185) and 66.5% (119/179) received testing by month 12, and 85.4% (158/185) and 87.2% (156/179) received testing by month 24, respectively. Kaplan-Meier curves demonstrating the time to HCV testing among all study participants, by randomization group, are presented in Figure 1.

**Figure 2:**
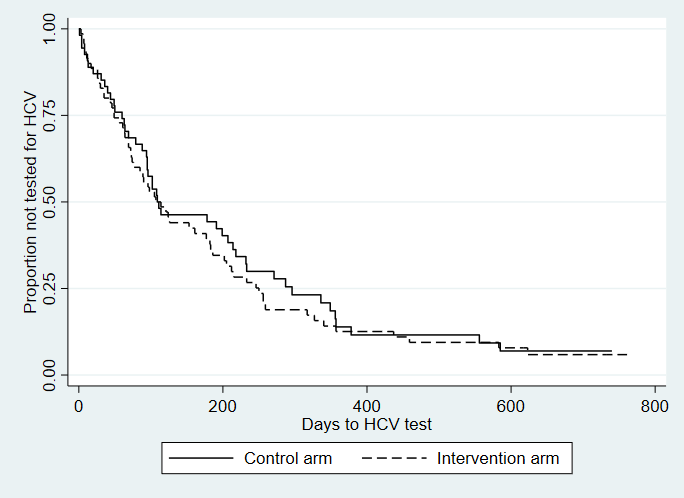
Time to hepatitis C virus test among people who injected drugs, by randomization group: Addiction-Comprehensive Health Enhancement Support System Study, 2016-2020. Hazard ratio: 1.34; 95% CI 0.87-2.05; *P*=.18; n=109. HCV: hepatitis C virus.

Of the 364 study participants who completed at least one follow-up interview, 34.1% (124/364) were considered to have injected drugs in the 4 months before either the baseline or any follow-up survey (70/185, 37.8%, in the intervention arm and 54/179, 30.2%, in the control arm). Of the 124 participants, 15 were excluded from the analysis because they did not report the risk of injecting drugs before undergoing HCV testing. Among the 109 participants who injected drugs and were included in the analysis (63 in the intervention arm and 46 in the control arm), 89% (56/63) of the participants in the intervention arm received an HCV test after injection reporting compared with 85% (39/46) in the control arm. Although not statistically significant, there is a trend toward an increased rate of HCV testing among intervention participants. Compared with those in the control group who injected drugs, the rate of HCV testing among those who injected drugs and used A-CHESS was 1.34 times higher (hazard ratio: 1.34; 95% CI 0.87-2.05; *P*=.18; Figure 2).

**Figure 3:**
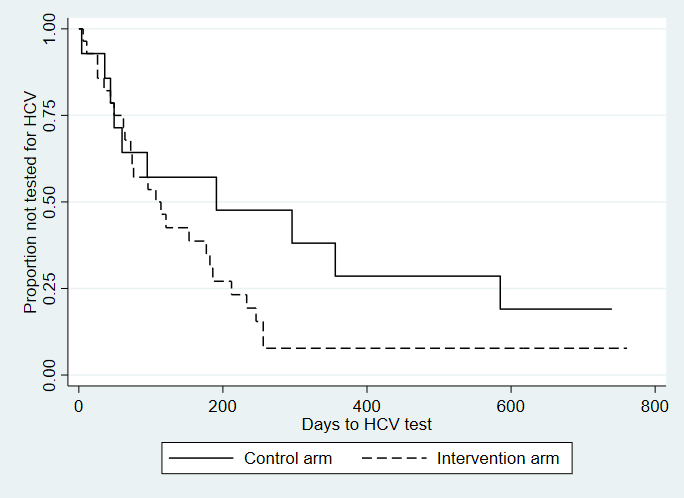
Time to hepatitis C virus test among people who shared injection equipment, by randomization group: Addiction-Comprehensive Health Enhancement Support System Study, 2016-2020. Hazard ratio: 2.92; 95% CI 0.959-8.86; *P*=.06; n=32. HCV: hepatitis C virus.

Of the 364 study participants who completed at least one follow-up interview, 11.5% (42/364) were considered to have shared injection equipment with another person in the 4 months before either the baseline or any follow-up survey (28/185, 15.1%, in the intervention arm and 14/179, 7.8%, in the control arm). Of the 42 participants, 10 were excluded from the analysis because they did not report the risk of sharing injection equipment before undergoing HCV testing. Among the 32 participants who shared injection equipment and were included in the analysis (23 in the intervention arm and 9 in the control arm), 87% (20/23) of the participants in the intervention arm received an HCV test after reporting sharing, compared with 56% (5/9) in the control arm. Further approaching statistical significance, the rate of HCV testing among those who shared equipment and used A-CHESS was 2.92 times higher than the rate among those in the control group who shared equipment (hazard ratio: 2.92; 95% CI 0.959-8.86; *P*=.06; Figure 3).

## Discussion

### Principal Findings

The goal of this study was to understand the HCV care continuum for people with OUD who are in early remission and receiving MAT and to determine whether implementing HCV-related content and functionality into the evidence-based A-CHESS system may improve HCV outcomes for this population. We found that 44.2% (184/416) of the participants were HCV Ab positive at baseline, indicating that nearly half of the study population had been exposed to the virus at some point before study enrollment. An overall improvement in the HCV care continuum between baseline and month 24 was observed, where a smaller proportion of participants were untested and a higher proportion had tested HCV negative and achieved SVR. These trends appeared among both intervention and control participants, suggesting that there may be external factors influencing changes in the HCV care continuum.

Among the entire study population, we did not observe a difference in HCV testing uptake between those who used A-CHESS and those who did not. However, when focusing on the subset who engaged in injection drug use, there was a slight trend toward increased HCV testing uptake among those who used A-CHESS (89% vs 85%), and a stronger trend was observed when focusing on those who reported sharing injection equipment with another person (87% vs 56%). These results suggest that A-CHESS may increase HCV testing rates if targeted at those with the highest risk of infection: those who share contaminated injection equipment. The intervention likely had no effect on HCV testing uptake among people who had not injected drugs because those participants are at a significantly lower risk of contracting HCV. Future studies that are powered to detect differences in HCV testing among these highest-risk groups are needed.

### Limitations

A limitation of this study was that the surveys asked if people injected drugs *in the 30 days before the survey* and shared injection equipment *in the 4 months before the survey*. The fact that these surveys did not ask whether individuals *ever* injected drugs or *ever* shared injection equipment, coupled with the fact that more participants were HCV Ab positive at baseline (n=184) than those who reported injecting drugs (n=124), suggests that we underestimated the number of participants who engaged in these behaviors and who may be at high risk for HCV. In addition, the number of people achieving subsequent steps of the HCV care continuum (eg, linkage to care, and treatment initiation) in this study, particularly among high-risk participants such as those who share injection equipment, limited our ability to test the effect of A-CHESS on these important stages of care.

The logic used to assign participants an HCV stage of care at each time point (Tables 1 and 2) allowed us to best characterize the HCV care continuum after each quarterly interview; however, this logic does have limitations because of the complex nature of the disease. Staging the disease in this manner did not allow us to estimate reinfection or HCV treatment failure experiences. In addition, this logic allows participants to advance to the latest stage, achieving SVR, during the study period if they had received treatment before the study but had not undergone RNA testing in the year before enrollment and then received an RNA negative test during the study period. Further analysis is required to estimate how many participants were cured during the study versus before the study.

Our team experiences enrollment challenges at 2 of the 3 addiction treatment centers, primarily because of limited staffing. Owing to these enrollment challenges, 97% of participants were recruited from 1 center in the state of Massachusetts, limiting the generalizability of this study. The primary treatment center is located in an urbanized area with a population size of approximately 89,000 residents [27], where HCV testing services may be more accessible than in rural communities. As suburban and rural communities have been disproportionately affected by the opioid crisis [28-30] and many HCV outbreaks concentrated in rural communities have been detected [31-33], understanding how A-CHESS serves communities with fewer resources for prevention and treatment is needed. Furthermore, only 2 participants were recruited in the state of Wisconsin. These participants may face unique barriers and facilitators to HCV testing compared with residents of Massachusetts; however, the results described in this paper did not change when the 2 Wisconsin residents were excluded.

Another limitation of this study is the use of self-report. The ability of HCV to spontaneously clear without antiviral therapy and the varying levels of clinical testing (eg, HCV Ab, HCV RNA, and tests to evaluate liver damage) complicates the disease, making it difficult for laypersons to understand their exact HCV stage of care. Prior studies have identified significant gaps in knowledge of HCV among people who inject drugs, particularly with respect to transmission risks, symptoms and clinical markers, and treatment options [34-36]. This study would have been strengthened if serologic confirmation of HCV could have been obtained. Likewise, participants often had difficulty remembering the exact date of their last HCV test. Consequently, most test dates were estimated.

### Strengths

As mobile health (mHealth) innovations continue to emerge and evolve, the opportunity to address complex, comorbid health conditions on a larger scale grows. To our knowledge, this is the first study to combine an addiction intervention with an HCV intervention to simultaneously address opioid dependence, risky injection behaviors, and HCV infection among people with OUD using a single mHealth app. In addition to the novelty of this intervention, many aspects of the design and analysis strengthened this study. By randomizing participants to the intervention and control arms, the chances of bias because of confounding factors and selection bias have been reduced. In addition, the intention-to-treat analysis maintains the advantages of random assignment and accounts for the fact that some participants assigned to the A-CHESS intervention may have used the app minimally or not at all.

### Conclusions

The prevalence of OUD and associated HCV infections has escalated in the United States. Advancements in mHealth technology offer the opportunity to provide information and services for the treatment of both OUD and HCV infection simultaneously. When implemented among populations who engage in high-risk behaviors such as sharing injection equipment, the HCV-enhanced A-CHESS intervention may increase awareness of HCV infection while preventing opioid relapse; however, studies that are powered to detect differences in HCV testing among high-risk groups are needed.

## Appendix

Multimedia Appendix 1 A comparison of baseline characteristics between the initial sample (N=416) and the final sample (n=266).

Multimedia Appendix 2 Percentage of individuals in each hepatitis C virus stage of care at baseline and each follow-up: Addiction-Comprehensive Health Enhancement Support System Study, 2016-2020. Ab: antibody; HCV: hepatitis C virus; SVR: sustained virologic response.

Multimedia Appendix 3 The hepatitis C virus care continuums for intervention and control groups at each time point.

Multimedia Appendix 4 CONSORT-EHEALTH checklist (V 1.6.1).

## Abbreviations

Ab: antibody
A-CHESS: Addiction-Comprehensive Health Enhancement Support System
HCV: hepatitis C virus
MAT: medications for addiction treatment
mHealth: mobile health
OR: odds ratio
OUD: opioid use disorder
RCT: randomized controlled trial
SVR: sustained virologic response
