# Supplementary material for: Effect of an mHealth Intervention on Hepatitis C Testing Uptake Among People With Opioid Use Disorder: Randomized Controlled Trial
Source: JMIR Mhealth Uhealth. 2021 Feb 22;9(2):e23080. doi: 10.2196/23080 (PMC7939944; doi:10.2196/23080)
Supplement: Multimedia Appendix 1 [file mhealth_v9i2e23080_app1.docx]

**Multimedia Appendix 1**

There were no significant differences in baseline characteristics between the initial sample (N=416) and the final, 24-month sample (N=266).

| **Characteristic** | **Category** | **Initial Sample**  **(N=416)** | **Final Sample (N=266)** | ***P*-value** |
| --- | --- | --- | --- | --- |
| Enrollment Site, n (%) | Wisconsin | 2 (0.05) | 2 (1) | 0.548 |
|  | Massachusetts- Clinic 1 | 9 (2) | 3 (1) |  |
|  | Massachusetts- Clinic 2 | 405 (97) | 261 (98) |  |
| Age, years, mean |  | 37 (10.0) | 38 (10.2) | 0.207 |
| Age of first opioid use, years, mean (st. dev) |  | 20 (7.2) | 21 (7.6) | 0.084 |
| Gender, n (%) | Male | 228 (54) | 143 (54) | 0.789 |
|  | Female | 188 (45) | 123 (46) |  |
| Race, n (%) | White | 409 (98) | 261 (98) | 0.849 |
|  | Non-White | 7 (2) | 5 (2) |  |
| Ethnicity, n (%) | Non-Hispanic/Latino | 377 (91) | 238 (90) | 0.655 |
|  | Hispanic/Latino | 38 (9) | 27 (10) |  |
| Highest education level, n (%) | Less than high school | 131 (31) | 79 (30) | 0.875 |
|  | HS diploma or GED | 162 (39) | 105 (39) |  |
|  | Some college or college degree | 123 (30) | 82 (31) |  |
| Currently employed, n (%) | Yes | 101 (24) | 66 (25) | 0.875 |
| Currently have a spouse/partner/significant other, n (%) | Yes | 238 (57) | 148 (56) | 0.686 |
| Diagnosed with HIV, n (%) | Yes | 4 (1) | 3 (1) | 0.833 |
| Diagnosed with mental health illness other than substance use disorder, n (%) | Yes | 293 (70) | 181 (68) | 0.509 |
| Current MAT, n (%) | Vivitrol | 24 (6) | 10 (4) | 0.428 |
|  | Suboxone | 90 (22) | 54 (20) |  |
|  | Methadone | 302 (73) | 202 (76) |  |
| Ever injected drugs, n (%) | Yes | 95 (36) | 49 (28) | 0.168 |
| Ever shared injection equipment, n (%) | Yes | 24 (6) | 9 (3) | 0.157 |
| Randomization group | Control | 207 (50) | 144 (54) | 0.265 |
|  | Intervention | 209 (50) | 122 (45) |  |
