# Supplementary figures and images for: Effect of an mHealth Intervention on Hepatitis C Testing Uptake Among People With Opioid Use Disorder: Randomized Controlled Trial
Source: JMIR Mhealth Uhealth. 2021 Feb 22;9(2):e23080. doi: 10.2196/23080 (PMC7939944; doi:10.2196/23080)

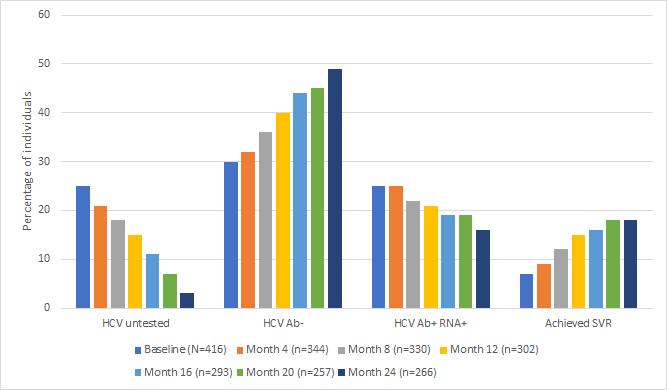

Supplement: Multimedia Appendix 2 [file mhealth_v9i2e23080_app2.png]
