# Supplementary material for: Effect of an mHealth Intervention on Hepatitis C Testing Uptake Among People With Opioid Use Disorder: Randomized Controlled Trial
Source: JMIR Mhealth Uhealth. 2021 Feb 22;9(2):e23080. doi: 10.2196/23080 (PMC7939944; doi:10.2196/23080)
Supplement: Multimedia Appendix 3 [file mhealth_v9i2e23080_app3.docx]

**Multimedia Appendix 2**

The HCV care continuums for intervention and control groups at each timepoint were comparable.

|  | **Baseline n (%)** | | **Month 4 n (%)** | | **Month 8 n (%)** | | **Month 12 n (%)** | |
| --- | --- | --- | --- | --- | --- | --- | --- | --- |
|  | **Intervention** | **Control** | **Intervention** | **Control** | **Intervention** | **Control** | **Intervention** | **Control** |
| **Total N** | 209 | 207 | 180 | 164 | 166 | 164 | 150 | 152 |
| **HCV Untested** | 55 (26) | 51 (25) | 42 (23) | 30 (18) | 32 (19) | 27 (16) | 21 (14) | 24 (16) |
| **HCV Ab-** | 59 (28) | 67 (32) | 54 (30) | 57 (35) | 55 (33) | 63 (38) | 58 (39) | 64 (42) |
| **HCV Ab+ no RNA test** | 13 (6) | 9 (4) | 3 (2) | 7 (4) | 1 (1) | 5 (3) | 0 (0) | 1 (1) |
| **HCV Ab+ RNA- (i.e. “cleared”)** | 9 (4) | 17 (8) | 10 (6) | 16 (10) | 9 (5) | 16 (10) | 11 (7) | 11 (7) |
| **HCV Ab+ RNA+** | 57 (27) | 46 (22) | 50 (28) | 35 (21) | 44 (27) | 28 (17) | 36 (24) | 28 (18) |
| **Currently on Treatment** | 2 (1) | 0 (0) | 7 (4) | 3 (2) | 4 (2) | 5 (3) | 2 (1) | 2 (1) |
| **Achieved SVR (i.e. “cured”)** | 12 (7) | 17 (8) | 14 (8) | 16 (10) | 21 (13) | 20 (12) | 22 (15) | 22 (14) |
|  | **Month 16 n (%)** | | **Month 20 n (%)** | | **Month 24 n (%)** | |  |  |
|  | **Intervention** | **Control** | **Intervention** | **Control** | **Intervention** | **Control** |  |  |
| **Total N** | 144 | 149 | 116 | 141 | 122 | 144 |  |  |
| **HCV Untested** | 17 (12) | 14 (9) | 9 (8) | 8 (6) | 2 (2) | 7 (5) |  |  |
| **HCV Ab-** | 56 (39) | 73 (49) | 46 (40) | 69 (49) | 55 (45) | 75 (52) |  |  |
| **HCV Ab+ no RNA test** | 0 (0) | 0 (0) | 1 (1) | 2 (1) | 3 (3) | 3 (2) |  |  |
| **HCV Ab+ RNA- (i.e. “cleared”)** | 11 (8) | 15 (10) | 9 (8) | 16 (11) | 12 (10) | 15 (10) |  |  |
| **HCV Ab+ RNA+** | 33 (23) | 22 (15) | 29 (25) | 20 (14) | 28 (23) | 15 (10) |  |  |
| **Currently on Treatment** | 2 (1) | 3 (2) | 1 (1) | 2 (1) | 0 (0) | 2 (1) |  |  |
| **Achieved SVR (i.e. “cured”)** | 25 (17) | 22 (15) | 21 (18) | 24 (17) | 22 (18) | 27 (19) |  |  |
